# Comparative Transcriptome Analysis Reveals the Interaction of Sugar and Hormone Metabolism Involved in the Root Hair Morphogenesis of the Endangered Fir *Abies beshanzuensis*

**DOI:** 10.3390/plants12020276

**Published:** 2023-01-06

**Authors:** Bin Liu, Ke Liu, Xiaorong Chen, Duohong Xiao, Tingjin Wang, Yang Yang, Hui Shuai, Sumei Wu, Lu Yuan, Liping Chen

**Affiliations:** 1 Department of Horticulture, College of Agriculture and Biotechnology, Zhejiang University, Hangzhou 310058, China; 2 Qingyuan Conservation Center of Qianjiangyuan-Baishanzu National Park, Qingyuan 323800, China

**Keywords:** *Abies beshanzuensis*, in vitro plantlets, comparative transcriptome, root hair morphogenesis, sugar metabolism, hormone signaling

## Abstract

*Abies beshanzuensis*, an extremely rare and critically endangered plant with only three wild adult trees globally, is strongly mycorrhizal-dependent, leading to difficulties in protection and artificial breeding without symbiosis. Root hair morphogenesis plays an important role in the survival of mycorrhizal symbionts. Due to the lack of an effective genome and transcriptome of *A. beshanzuensis*, the molecular signals involved in the root hair development remain unknown, which hinders its endangered mechanism analysis and protection. Herein, transcriptomes of radicles with root hair (RH1) and without root hair (RH0) from *A. beshanzuensis* in vitro plantlets were primarily established. Functional annotation and differentially expressed gene (DEG) analysis showed that the two phenotypes have highly differentially expressed gene clusters. Transcriptome divergence identified hormone and sugar signaling primarily involved in root hair morphogenesis of *A. beshanzuensis*. Weighted correlation network analysis (WGCNA) coupled with quantitative real-time PCR (qRT-PCR) found that two hormone–sucrose–root hair modules were linked by *IAA17*, and *SUS* was positioned in the center of the regulation network, co-expressed with *SRK2E* in hormone transduction and key genes related to root hair morphogenesis. Our results contribute to better understanding of the molecular mechanisms of root hair development and offer new insights into deciphering the survival mechanism of *A. beshanzuensis* and other endangered species, utilizing root hair as a compensatory strategy instead of poor mycorrhizal growth.

## 1. Introduction

*Abies beshanzuensis* M. H. Wu, an important fir of the Pinaceae family, is a relic plant from the quaternary glacial period of ancient origin. It has a reputation as a “living fossil” and has been designated as one of the 12 global critically endangered plant species listed by the International Union for Conservation of Nature (IUCN) since 1987 [1]. Additionally, it was recorded in the China Plant Red Data Book [2] and has been subsequently categorized at ‘first-grade’ for national protection since 1999 [3]. *A. beshanzuensis* is shade-tolerant and prefers cold and humidity, only distributed on the main peak of Mt. Baishanzu of China at an altitude of 1740–1750 m, which may be evidence that psychrophytes in low latitudes retreated to high altitudes at the end of the quaternary glacial period. Unfortunately, there are only three wild adult trees of *A. beshanzuensis* remaining in the world at present, and they are considered critically endangered. Therefore, it is urgent to preserve its germplasm resources, analyze the endangered mechanisms and expand its narrow habitat.

In recent years, plentiful measures have been taken to rejuvenate the mother tree and artificially breed seedlings of *A. beshanzuensis* due to its sparse individual population and extremely weak natural regeneration ability [4]. However, the number of artificially bred seedlings is still small, and long-term natural reproduction and conservation remain unresolved. Hence, it is necessary to imminently use plant tissue culture and other technical methods to assist the breeding of *A. beshanzuensis*. However, *A. beshanzuensis* belongs to a strong mycorrhizal symbiosis-dependent gymnosperm, which primarily relies on mycorrhiza for the absorption and utilization of nutrients to undergo normal growth and development [5,6]. Departing from the mycorrhizal symbiosis in its natural state will lead to growth and development obstacles or even death of *A. beshanzuensis*, whereas high habitat elevation results in the imbalance of rhizosphere microbial relationship, which seriously hinder the nutrient absorption, growth and development [5,7]. In the early stage, our team has made a breakthrough in obtaining plantlets of *A. beshanzuensis* in vitro using immature embryo rescue technology, and we have established an artificial breeding system [8,9]. Importantly, *A. beshanzuensis* test-tube plantlets could grow in a sterile environment without mycorrhiza, but during transplanting and domestication, some plantlets cannot absorb nutrients normally, which makes it difficult for test-tube seedlings to return to their habitat. Overcoming the nutrient absorption problem after breaking out of the strongly dependent mycorrhizal symbiosis and exploring the regulatory mechanism in *A. beshanzuensis* is the key to save the precious endangered plant resources and solve their survival and reproduction problems.

Plant growth and development predominantly depend on root nutrient uptake [10,11]. Root hairs, epidermal protuberances from the root, constitute almost 90% of the surface area of the root system and expand the plant’s interface with the soil environment, which is beneficial in improving the absorption capacity for soil nutrient uptake [12,13]. Longer and/or denser root hairs can also enhance the water and mineral absorption [14,15,16,17]. However, *A. beshanzuensis* strongly depends on ectomycorrhizal symbiosis to complete normal growth and development, metabolism and other physiological activities [6], and their root hairs are extremely rare in nature. During the sterile cultivation process, we found that several test-tube plantlets of *A. beshanzuensis* appeared in two states, that is, radicles with root hair (RH1) and radicles without root hair (RH0). Additionally, the growth and development of test-tube plantlets with root hairs were significantly improved compared with the ones without root hairs after further transplantation. Dynamic morphogenesis of root hair is a vital trait for the plants to improve the acquisition of essential nutrients and growth development, especially for the endangered species.

It is essential that we improve comprehension of the root hair developmental pathway. Epidermal cells in the mature zone of plant roots include non-rooting hair cells (N, atrichoblast) and rooting hair cells (H, trichoblast). The process of root hair growth and development can be roughly divided into four stages: root hair cell fate determination, root hair initiation, root hair apical growth, and root hair maturation [18,19,20]. Initiation and elongation of root hair and tip growth are controlled by many different, yet interconnected, molecular pathways [20,21]. This regulation framework includes complex interactions of some hormones (auxin, ethylene, cytokinin, etc.) and nutrients (sugars). Auxins are involved in regulating the morphogenesis of root hair, which can change the root hair length or even restore root hair growth in the hairless mutants [22,23]. Relative transcriptional activities of *AUXIN RESPONSE FACTORS* (*ARFs*) can both suppress and improve the growth of root hair [24,25]. Auxin signals are commonly interacted with ethylene to regulate root hair development. Ethylene can participate in the epidermal cell fate determination process, such as activating key root hair genes’ expression, such as *RHD6*, *RSL2* and *RSL4*, to regulate root hair initiation [20,26,27,28]. Additionally, the crosstalk between auxin, ethylene, cytokinin and other hormonal signals is instrumental in the root hair developmental pathway, which controls root hair morphogenesis [29,30,31].

In addition, root growth and branching patterns are closely related to sugar supply [32,33,34]. Sucrose can induce the auxin response pathway by activating *PIN* transporters and the stem cell niche, promoting the division and elongation of root cells in WT seedlings and *med12* and *med13* mutants of *Arabidopsis thaliana* [33,35,36]. Many pivotal genes such as *IAA17*, *OXI*, *VLN4* and *XIK*, regulating the initiation and elongation of root hair and their regulation mechanisms by hormone and sugar cues, have been discovered in *Arabidopsis* [37,38,39] and monocot species based on transcriptomics, single-cell RNA sequencing and other molecular biology methods [20,40,41]; however, relevant reports are still unavailable in the endangered fir.

Root hair may be an essential survival strategy for *A. beshanzuensis* after dispensing with their ‘symbiosis partner’. However, the regulation mechanism on the growth and development of root hair from *A. beshanzuensis* remains unknown. In this study, we performed transcriptome sequencing throughout root hair development in RH1 and RH0 and identified the key gene co-expression modules related to hormone and sugar metabolism involved in the root hair morphogenesis. Our study will help to illustrate the molecular mechanisms of root hair development in *A. beshanzuensis* to further study the survival mechanism of *A. beshanzuensis* and other endangered species dependent on mycorrhiza.

## 2. Results

### 2.1. Comparison of Morphology from Test-Tube Plantlets of A. beshanzuensis In Vitro Conditions

**Figure 1:**
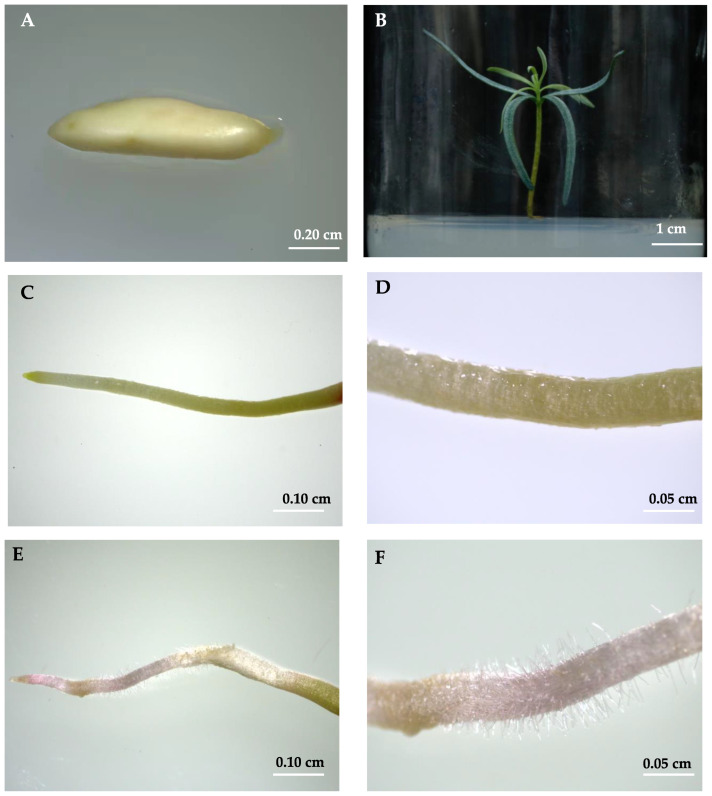
Phenotypes of test-tube plantlets of *A. beshanzuensis*. (**A**) Immature embryo of *A. beshanzuensis*. Bar, 0.20 cm. (**B**) The test-tube plantlet of *A. beshanzuensis*. Bar, 1 cm. (**C**) Radicles without root hair of *A. beshanzuensis*. Bar, 0.10 cm. (**D**) Zoom of radicles without root hair of *A. beshanzuensis*. Bar, 0.05 cm. (**E**) Radicles with root hair of *A. beshanzuensis*. Bar, 0.10 cm. (**F**) Zoom of radicles with root hair of *A. beshanzuensis*. Bar, 0.05 cm.

To protect *A. beshanzuensis* from becoming severely endangered, plant tissue culture, such as embryo culture and somatic embryogenesis, is an indispensable and efficient measure to expand its population range. Immature seeds of *A. beshanzuensis* were collected and plump embryos were isolated with a sterile operation, before being inoculated on the DCR medium (containing sucrose (20 g·L^−1^), hydrolyze casein (500 mg·L^−1^) and agar (8 g·L^−1^), pH 5.8) to develop test-tube plantlets of *A. beshanzuensis* (Figure 1A,B). The radicles appeared after seven days of culture under weak light conditions, and interestingly, morphological differences were found that the root hair developed on the cocked radicles, while nearly no root hair developed on the radicles attached to the medium (Figure 1C–F). Additionally, during the following transplant process, the test-tube plantlets of *A. beshanzuensis* with root hairs grew better than those without root hair. Hence, it is indicated that root hair might play a pivotal role in the growth and development of the endangered *A. beshanzuensis* free of mycorrhizal symbiosis.

### 2.2. RNA-Seq Sequencing Analysis and Functional Annotation

**Figure 2:**
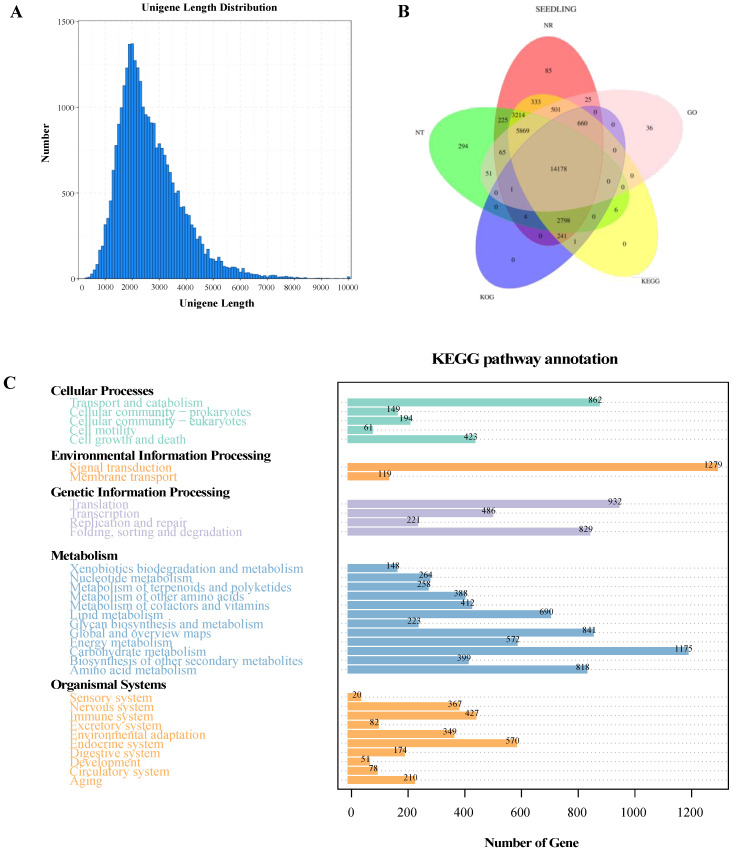
Annotation and classification of assembled unigenes in radicles development of *A. beshanzuensis*. (**A**) Length distribution of subreads at different development stage of two-type roots (RH1_B, RH1_0, RH1_A, RH0_B, RH1_0, and RH1_A), RH1, radicles with root hair; RH0, radicles without root hair; B represents 2 days before root hair initiation; 0, root hair initiation; A represents 2 days after root hair initiation, the same meaning as below. (**B**) A total of 28,923 unigenes were totally annotated using different protein databases. (**C**) KEGG assignments for functional classification of the assembled unigenes.

To find out the gene-expression modes responding to the developmental process of root hair from *A. beshanzuensis*, RNA-seq of different development stage of two-type roots (RH1_B, RH1_0, RH1_A, RH0_B, RH1_0, and RH1_A) was adopted to demonstrate the transcriptional gene expression profiles. Three biological replicates were employed at each time point. Eighteen sample RNA-Seq libraries were constructed and sequenced in total. According to the 95% similarity between sequences, the corrected transcript sequences have been clustered to remove the redundancy, and the length and frequency distribution of transcripts before and after the removal of redundancy were statistically analyzed (Figure 2A). A total of 83,384 transcripts and 28,923 unigenes were assembled, and the sequence length distribution after redundancy removal was counted (Tables S3 and S4).

To obtain comprehensive gene function information and annotate the gene function of the de-redundant sequences using CD-HIT software, the databases used include Nr, Nt, Pfam, KOG/COG, Swiss-prot, KEGG, and GO. Among them, 28,199 unigenes have been annotated to the Nr protein database, 26,705 unigenes have been annotated to the Nt database, 21,386 unigenes have been annotated to the Pfam database, 21,387 unigenes have been annotated to the GO database, 27,070 unigenes have been annotated to the KOG/COG database, 24,662 unigenes have been annotated to the Swiss-prot database, and 27,801 unigenes have been annotated to the KEGG database (Figure 2B,C and Figure S1). We classified the functions of all predicted unigenes using GO and KEGG assignments. The GO terms “metabolic process (GO:0008152)”, “cellular process (GO:0009987)”, and “single-organism process (GO:0044699)” were enriched in the biological process category. The GO terms “cell (GO:000562)”, “cell part (GO:0044464)”, and “organelle (GO:0043226)” were enriched in the cellular component category. The GO terms “binding (GO:0005488)”, “catalytic activity (GO:0003824)”, and “transporter activity (GO:0005215)” were enriched in the molecular function category (Figure S1). Additionally, the most enriched KEGG pathways were “signal transduction (ko04016)”, “carbohydrate metabolism (ko01212)”, “translation (ko03013)”, and “transport and catabolism (ko04138)” (Figure 2C).

### 2.3. Comparative Analysis of DEGs during the Development of the A. beshanzuensis Root with/without Root Hair

**Figure 3:**
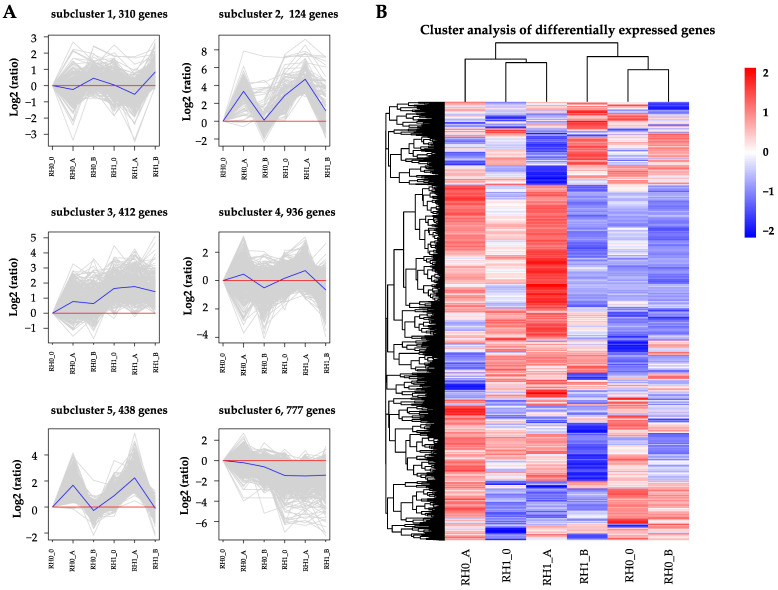
Expression patterns of differentially expressed genes (DEGs) at different development stages of two-type roots in *A. beshanzuensis*. (**A**) K-means clustering of the identified DEGs at different development stage of two-type roots in *A. beshanzuensis*. All the identified DEGs were classified into 6 clusters. (**B**) Cluster analysis of the identified DEGs at different development stage of two-type roots in *A. beshanzuensis*.

In total, 4839 DEGs were identified different at development stages of two-type roots (RH1_B, RH1_0, RH1_A, RH0_B, RH1_0, and RH1_A). To analyze the expression patterns of identified DEGs, k-means clustering was conducted (Figure 3A). The result showed that the 4839 DEGs were classed into six clusters that exhibited distinct expression modes in RH0 and RH1. In general, clusters 2, 4 and 5 exhibited similar expression patterns during the three stages of root hair development, and only clusters 1, 3 and 6 displayed relatively disparate expression patterns. Notably, the DEGs in cluster 3 were consistently up-regulated at the three development stages in RH1 compared to RH0. On the contrary, the DEGs in cluster 6 showed down-regulated expression patterns at the three development stages in RH1 compared to RH0. Cluster analysis showed that the unigenes had diverse expression patterns and could be divided into six classes, containing unigenes highly expressed in RH1 and RH0 (Figure 3B). The finding indicated that these DEGs played different roles during the root development of *A. beshanzuensis*.

### 2.4. Identification of DEGs of Hormone Signals Involved in Root Hair Growth and Development

**Figure 4:**
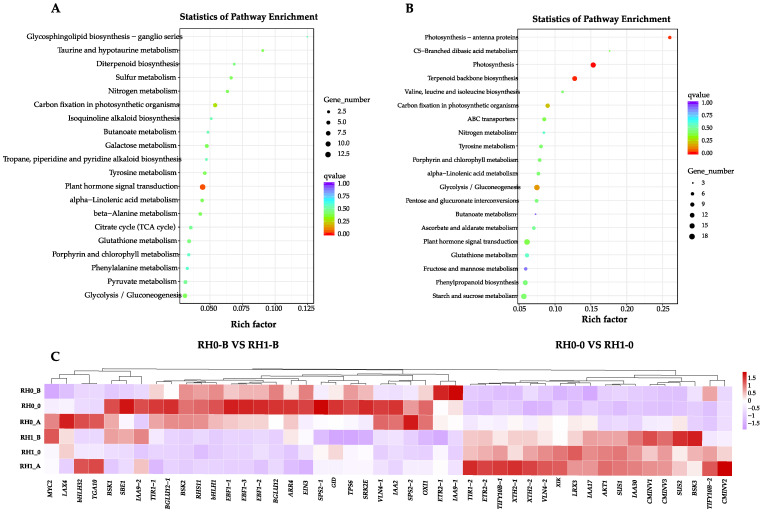
Identification of DEGs involved in hormone and sugar signal transduction at different development stages of two-type roots in *A. beshanzuensis*. (**A**) Identification of DEGs involved in hormone signal transduction in groups of RH0_B, RH0_0, RH1_B and RH1_0 from *A. beshanzuensis*. (**B**) Identification of DEGs involved in sugar pathway in groups of RH0_B, RH0_0, RH1_B and RH1_0 from *A. beshanzuensis*. (**C**) Cluster expression patterns of important DEGs involved in pathway of sugar, hormone and root hair growth and development in groups of RH1_B, RH1_0, RH1_A, RH0_B, RH1_0, and RH1_A of *A. beshanzuensis*.

Since the stages of root hair cell fate determination and root hair initiation were indispensable for the lifespan of root hair, we screened the DEGs in groups of RH0_B, RH0_0, RH1_B and RH1_0. Based on the GO and KEGG enrichment analysis, the largest number of DEGs were identified in the hormone signal transduction and the starch and sucrose metabolism pathway. We performed the cluster expression pattern analysis and found that genes of the hormone signal transduction and the starch and sucrose metabolism pathway involved in root hair morphogenesis showed different expression patterns (Figure 4C).

**Figure 5:**
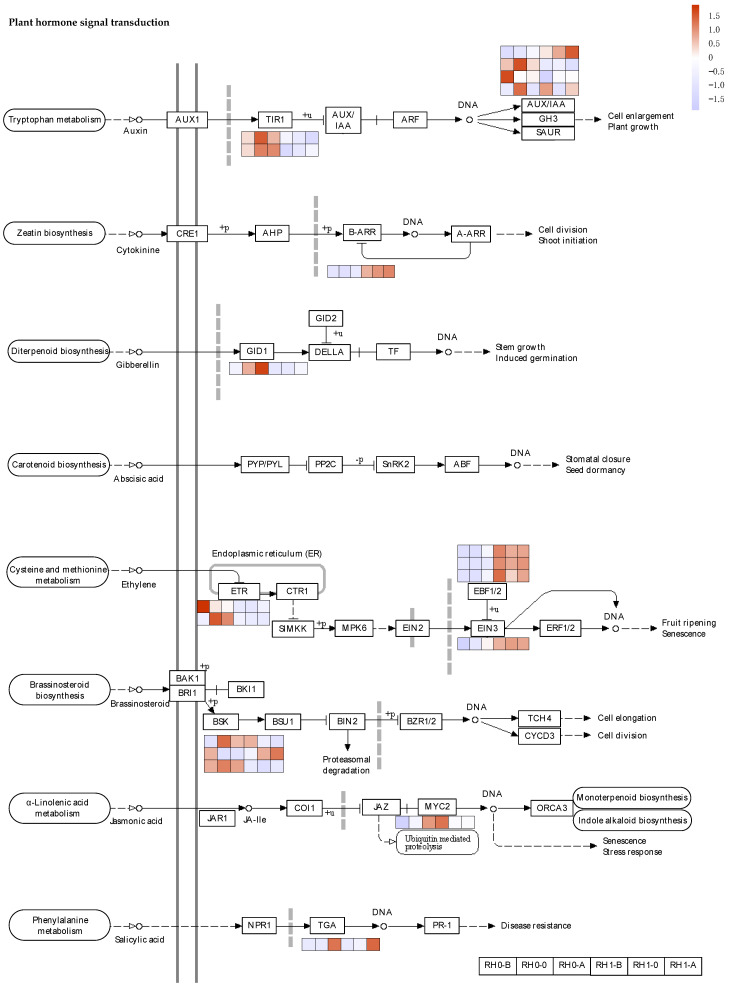
Expression pattern of key DEGs in hormone signal transduction at different development stage of two-type roots in *A. beshanzuensis*.

To investigate the genes involved in hormone signal transduction, we filtered our RNA-seq datasets. A total of 26 key regulators were identified in the hormone signaling pathway (Figure 4A). In the auxin signaling pathway, genes of *TIR1* and *AUXIAA* were differently expressed in the RH0 and RH1 group, which may be involved in cell enlargement and plant growth. *TIR1* genes (transcript44569/f5p0/2291 and transcript33559/f3p0/2760) were significantly upregulated in the RH0 group compared with RH1 group. Additionally, it showed a trend of rising first and then falling in the different development stages of the RH0 group, while in the RH1 group, it showed a gradual descent (Figure 5). In the cytokinine signaling pathway, *B-ARR* (transcript71841/f2p0/1603) was significantly up-regulated in the RH1 group and increased gradually during the root hair development process, which was defined to regulate cell division and shoot initiation. In addition, in the ethylene signaling pathway, the *ETRs* (transcript18258/f2p0/3462 and transcript17218/f20p0/3444) were highly expressed in the RH0 group, while *EBF1/2* (transcript29821/f2p0/2902, transcript29821/f2p0/2902, and transcript21823/f3p0/3265) and *EIN3* (transcript38824/f2p0/2633) were up-regulated in the RH1group compared with the RH0 group. Furthermore, in the brassinosteroid signaling pathway, *BSKs* (transcript26938/f2p0/3018, transcript40961/f3p0/2493, and transcript30921/f2p0/2859) were differently expressed in the two groups, which may be involved in cell elongation and division (Figure 5). Among them, transcript26938/f2p0/3018 decreased gradually during the development in the RH1 group; it was even silent after the root hair initiation, while transcript40961/f3p0/2493 showed a contrary trend under the same conditions. This finding indicated that dynamic hormone signaling may drive the morphology, initiation, growth and development of root hair.

### 2.5. Identification of DEGs of Sugar Metabolism Involved in Root Hair Growth and Development

**Figure 6:**
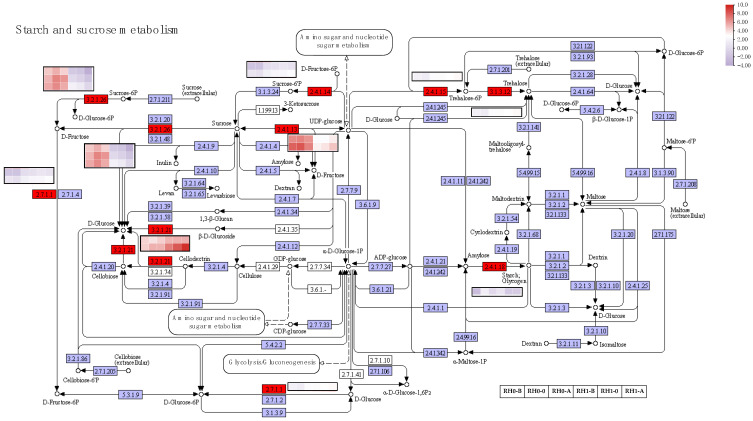
Expression pattern of key DEGs in sugar metabolism pathway at different development stage of two-type roots in *A. beshanzuensis*.

There were also plenty of DEGs enriched in the starch and sucrose metabolism pathway in our RNA-seq datasets. According to the KEGG annotated gene set, a total of 15 key genes were screened in the starch and sucrose metabolism pathway (Figure 4B). It was observed that 3 key genes of *CMINVs* (EC: 3.2.1.26, transcript57112/f3p0/2016, transcript57359/f5p0/2007, and transcript61228/f3p0/1906) were annotated in D-glucose-6P and D-fructose synthesis, which were significantly up-regulated in the RH0 group compared with the RH1 group. Specifically, these genes showed an earlier increase and later decrease trend in the different development stages of the RH0 group, but gradually decreased in that of the RH1 group. Meanwhile, 2 *SPS2* crucial genes (EC: 2.7.1.1, transcript4562/f2p0/4657 and transcript4764/f5p0/4651) annotated in D-fructose-6P synthesis and one gene (EC: 2.7.1.1, transcript4937/f7p0/4584) annotated in D-glucose synthesis were significantly highly expressed in RH1 group, which showed a gradual upward trend. In addition, 2 *BGLU12* pivotal genes (EC: 3.2.1.21, transcript62004/f3p0/1885 and transcript62679/f2p0/1894) annotated in D-glucose and cellobiose synthesis, showed significant up-regulated during the development of the RH1 group. They increased gradually, and transcript62679/f2p0/1894, specifically, rose by 17-fold in the later development stage of RH1 group higher than that of RH0 group. In total, 2 key *SUSs* (EC: 2.4.1.13, transcript21012/f2p0/3294 and transcript20257/f62p0/3275) annotated in the sucrose and UDP-glucose synthesis and decomposition process were expressed significantly higher in the RH0 group compared with the RH1 group. They showed an earlier increase and later decrease in the different development stages of RH0 group; in particular, transcript20257/f62p0/3275 increased by nearly 20-fold at RH0-B compared with RH1-B (Figure 6). The *SPS2* (EC: 2.4.1.14, transcript4562/f2p0/4657 and transcript4764/f5p0/4651) annotated in sucrose-6P synthesis was up-regulated during the development of the RH1 group and was almost not expressed in that of the RH0 group. *TPS6* (EC: 2.4.1.15, EC: 3.1.3.12 and transcript4937/f7p0/4584) annotated in trehalose synthesis was significantly up-regulated during the development of the RH1 group and increased gradually. These results suggested that sucrose metabolism pathway signaling may be bound to the root system architecture, in particular, root hair initiation in *A. beshanzuensis*.

### 2.6. Interaction Analysis of Sucrose and Hormones Metabolism Signals Involved in Root Hair Development

**Figure 7:**
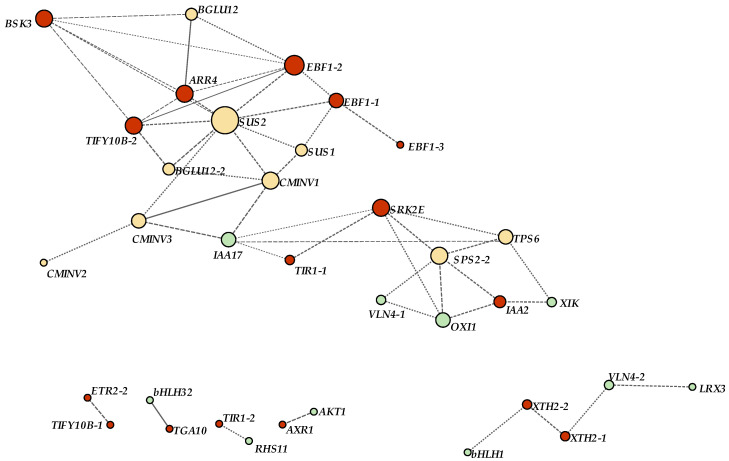
Screening of hub DEGs in hormone signal transduction and sugar metabolism pathway related to root hair growth and development genes in root development regulation of *A. beshanzuensis*. Each node indicates a gene. Yellow nodes, hub DEGs in sugar metabolism pathway; red nodes, hub DEGs in hormone signal transduction; green nodes, gene connections involved in root hair growth and development of *A. beshanzuensis*. Bigger nodes indicate more connections.

In order to further study the regulation relationship between sucrose, hormones, and root hair, the genes related to root hair development reported in the literature were screened, such as *IAA17*, *XIK*, *VLN4*, *BHLH32*, *LRX3*, and *OXI1* [20,38], and as detailed in Table S2. These gene sequences were subjected to BLASTN searches against the transcriptome sequences to identify homologous sequences in *A. beshanzuensis* with E values < 10-10. We screened the homologous root hair development genes with the best alignment rate in *A. beshanzuensis* used for WGCNA (Table S2). To further investigate the hub regulators in the sucrose and hormone metabolism signal pathways during the root hair development of *A. beshanzuensis*, WGCNA was performed to parse the interactional network of DEGs (Figure 7). In the root hair gene module, *IAA17* is a negative regulator for root hair development, which caused a reduction in root hair in *Arabidopsis* [42]. In contrast, *OXI*, *VLN4* and *XIK* are defined as the key factors to promote root hair elongation and tip growth in *Arabidopsis* [20,37,38]. The results found that two hormone–sucrose root hair modules were linked by *IAA17*, which led to *SUS2*, a hub gene co-regulated by sucrose and hormone signals, being co-expressed with *ARR4*, *TIFY10B*, *EBF1*, and *BSK3* in the hormone signal transduction pathway, *BGLU12* and *CMINV* in the sucrose synthesis pathway, and *IAA17*, *OXI*, *VLN4* and *XIK* in root hair regulation. These results suggest that the sucrose pathway is co-regulated with hormones such as auxin, cytokinin and ethylene, and *SUS2* was positioned in the center of the regulation network. At the same time, *SRK2E*, as a hub gene in hormone signal transduction, was co-expressed with *SPS*, *OXI*, *VLN4* and *XIK* genes to regulate root hair length (Figure 7).

### 2.7. qPCR Validation for Reference Genes and DEGs of Sucrose and Hormones Signals Involved in Root Hair

**Figure 8:**
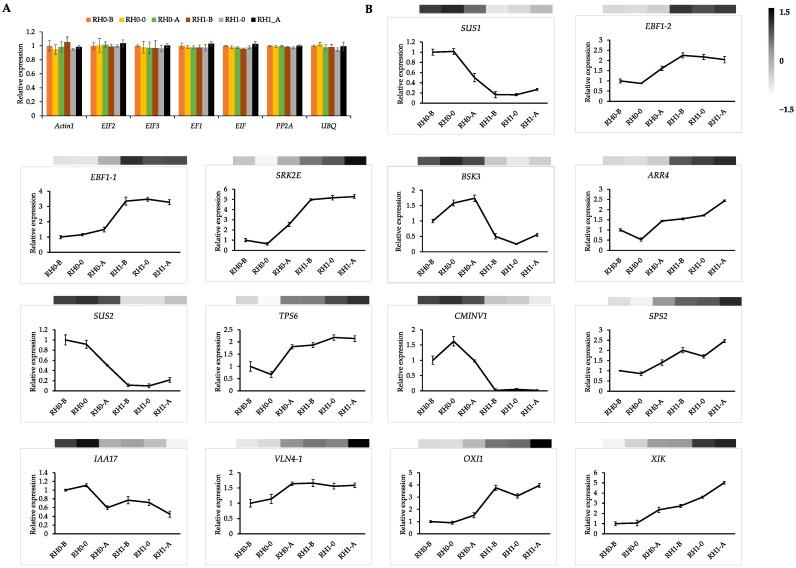
Expressions of 14 DEGs in hormone and sugar signal involved in root hair growth and development of *A. beshanzuensis* determined by qRT-PCR. (**A**) Expression stability of candidate reference genes of *A. beshanzuensis* determined by qRT-PCR. (**B**) Expressions of 14 DEGs in hormone and sugar signal involved in root hair growth and development of *A. beshanzuensis* determined by qRT- PCR. Error bars, standard deviation (SD) from three biological repeats.

Since few housekeeping genes have been reported in *A. beshanzuensis*, we firstly screened the expressional stability of reference genes. Difference in cycle threshold and variance coefficient were calculated to evaluate the expression stability of candidate genes. Seven candidate reference genes were evaluated in order to screen the superior internal reference genes for data normalization. *PP2A*, *EIF2*, *EIF3*, *EF1*, *EIF*, *UBQ* and *act1* were selected due to their stable expressions across eighteen sample pools, and *PP2A* showed the most expressional stability in different tissues and different root developmental stages (Figure 8A).

To validate the gene expression involved in sugar metabolism, hormone signaling and root hair pathways, qRT-PCR assays were performed on the samples of roots at different development stages from *A. beshanzuensis* plantlets. We selected five sugar-related genes, five hormone-related genes, and four root hair-related genes, including *SUS2*, *SRK2E* and *IAA17*, to verify our RNA-seq data. The specific primer sequences are listed in Table S1. The results showed that the expression of these chosen genes was basically consistent with RNA-seq results and showed a correlation of more than 0.9 (Figure 8B), which also indicated the reliability of our transcriptome. At the same time, further analysis of the qRT-PCR showed that *VLN4*, *OXI1* and *XIK*, as the key factors to promote root hair growth, were significantly higher in RH1-B and RH1-0 than that in RH0-B and RH0-0. On the contrary, *IAA17* acted as a negative regulator and was significantly higher in RH0-B and RH0-0 than in RH1-B and RH1-0. The co-expressed key genes in the sucrose pathway (*SUS2*, *SUS1* and *CMINV1*) and hormone signal regulator (*BSK3*) were significantly higher in RH0-B and RH0-0 than in RH1-B and RH1-0, showing a similar expression trend to *IAA17*. In addition, hormone signal regulators such as *ARR4* and *EBF1* were consistent with the expression trend of the *VLN4*, *OXI1* and *XIK*. This suggests that the above genes could be used as the candidate regulators of root hair morphogenesis.

## 3. Discussion

*A. beshanzuensis* is a strong mycorrhizal symbiosis plant, which hardly use roots to absorb and utilize nutrients to complete the normal growth and development without symbiosis or with poor symbiosis growth, leading to difficulties in artificial breeding. Root hair is an essential part of root, and its dynamic modifications play critical roles in nutrient utilization and adversity stress response. Root hair morphogenesis regulation involved in complicated signaling pathways, such as hormone transduction, sugar metabolism, protein interactions, and transcription factors, etc., whereas how the molecular signaling involved in the root hair growth and development of conifers is dependent on mycorrhizal symbiosis remains unknown.

### 3.1. Root Hair Acts as a Pivotal Survival Strategy for A. beshanzuensis Breaking out of the Strong-Dependent Mycorrhizal Symbiosis

Root hairs help plants absorb water and nutrients, anchor the root into the soil, and provide locations for the interactions with soil microorganisms [16,43,44]. In this study, we found that root hair of *A. beshanzuensi* developed on the cocked radicles (Figure 1E,F), while nearly no root hair on the radicles attached to the medium (Figure 1C,D). It can be hypothesized that the cocked radicles in the air under septic conditions might suffer from the adversity and lack water and nutrient elements, which promotes the root hair initiation for continue survival. Under nutrient-deficient conditions, nutrient accumulation was positively correlated with root hair length. Electrophysiological studies have found that root hairs can directly promote the absorption of nutrient elements [45]. The transport system operates on the root hair plasma membrane [17]. The length and density of root hairs can sense nutrient and water status in the soil, thereby regulating plant root growth and development and morphogenesis in response to environmental changes [45,46]. In the later stage of root system development, root hairs can participate in the formation of root sheaths and improve the ability of plants to adapt to stresses, thereby reducing the loss of water and nutrients [47].

Our group found that the root hair played an important role in the cultivation of test-tube plantlets of *A. beshanzuensis* under sterile conditions. Mycorrhizal fungi and root hairs are closely related as two methods for roots to obtain resources. It is generally believed that these two absorption strategies are complementary; that is, the role of mycorrhizal fungi can be replaced by root hairs and vice versa [48].

The habitat of *A. beshanzuensis* is at a high elevation where microbial activity is relatively weak, leading to a thick litter layer and an imbalance of a rhizosphere microbial relationship, which may seriously hinder the growth of *A. beshanzuensis*. In this case, the occurrence of root hair may serve as a compensatory mechanism to compensate for the decreased absorptive capacity associated with the reduced rate of fungal infection [15], which can serve as a survival strategy for *A. beshanzuensis*. Hence, it is crucial to determine the regulation mechanism of *A. beshanzuensis* root hair architecture.

### 3.2. Root Hair Morphogenesis of A. beshanzuensis Can Be Regulated by Hormone and Sugar Signaling

Whether or not an epidermal cell will mature into a root hair depends on a variety of factors, including outer environmental circumstances and various autologous transduction signals [20,49]. The *TRANSPORT INHIBITOR RESISTANT1/AUXIN SIGNALING F-BOX* (*TIR1/AFB*) receptor complex binding to the auxin contributes to the interplay between *TIR1/AFB* and *AUXIN/INDOLE-3-ACETIC ACID* (*Aux/IAA*) co-repressors, leading to *Aux/IAA* polyubiquitination and degradation [50,51]. In *Arabidopsis thaliana*, three *ARFs* (*ARF5*, *ARF7*, and *ARF8*) have been characterized to promote root hair growth, while eight root-hair-specific *ARFs* (*ARF1-4*, *ARF19-11*, and *ARF16*) were determined to suppress root hair growth [24,25]. Some auxin-signaling mutants, such as the *SHORT HYPOCOTYL 2* (*SHY2/IAA3*) mutant results in root hair extension [52]. In this study, *TIR1* and *AUX/IAA* of *A. beshanzuensis* were significantly differently expressed in the RH0 and RH1. They were significantly up-regulated in the RH0 group compared with the RH1 group (Figure 5), indicating that auxin signaling regulates root hair growth development. Type-B *ARABIDOPSIS RESPONSE REGULATORS* (*ARRs*) can control the cytokinin signaling through positive feedback regulation [28,53]. Additional cytokinin caused a root hair length increase in the wild-type *Arabidopsis*, and lowering the content of the endogenous cytokinin shortened root hair length [28]. Here, *B-ARR* of *A. beshanzuensis* was significantly up-regulated in the RH1 and increased gradually during the root hair development process (Figure 5), which showed that cytokinin signaling may be positively involved in root hair growth. Ethylene biosynthesis intercept or *ETR1* and *EIN2* function hinders root hair growth [54,55]. Additionally, ethylene activates the expression of crucial root hair genes, such as *RHD6, RSL2, RSL4,* and the *bHLH* transcription factor of the RSL family, which regulate the root hair initiation and tip growth of roots [27,28,30,56]. In our study, the *ETRs* were highly expressed in the RH0 group, while *EBF1/2* and *EIN3* were up-regulated in the RH1group. Exogenous glucose and sucrose can promote the growth of plant primary roots and the growth of the meristem, while high concentration of glucose can inhibit the growth of primary roots, and the number of lateral roots and root hairs changed when treated with different concentrations [6,57] found that light and sucrose had antagonistic effects, which jointly affected the occurrence and elongation of root hair. In this study, a total of 15 key genes were screened in the starch and sucrose metabolism pathway (Figure 4B), and two key *SUSs* were expressed significantly higher in the RH0 group, which showed an earlier increase and later decrease in the different development stages, while the *SPS2* genes were up-regulated during the development of the RH1 group, and were almost not expressed in that of the RH0 group (Figure 6). This suggests that hormones and sucrose signals may be bound to the root hair morphogenesis in *A. beshanzuensis*.

### 3.3. The Interaction of Hormone and Sugar Metabolism Pathway Plays an Indispensable Role in Root Hair Growth and Development

The interaction of various signals is crucial in the root hair morphogenesis of plants [20]. It has been illustrated that ethylene can activate auxin biosynthesis during root hair initiation in root tips [58,59,60]. In the *aux1-ein2* mutant, the root hair displayed a deficient phenotype, and numerous root hair genes were up-regulated by both auxin and ethylene signaling [55,61]. In addition, the hairless phenotype of *rhd6* root could be compensated by auxin and ACC involved in ethylene synthesis [22,28]. Auxin and ethylene may interact with *RHD6* to regulate downstream genes, such as *RSL1/2/4*, of the core-controlled root hair pathway [20,60]. Several primary genes in the network of root hair formation can also be targeted by auxin, ethylene, and cytokinin. For instance, all the three hormones (auxin, ethylene, cytokinin) could repair the *rhd6* mutant phenotype [28]. In addition, the auxin response factors of *ARF5* and *ARF7* could up-regulate *CYTOKININ RESPONSE FACTOR* (*CRF*) genes to participate in cytokinin biosynthesis [62,63] dditionally, it has been illustrated that cytokinin can also take part in ethylene biosynthesis by promoting ACC synthase stability [64]. Furthermore, strigolactones coupled with auxin play additive roles in root hair development [65] and regulate root hair elongation by activating ethylene signals related to auxin signals, which are involved in the root hair lifespan [66]. Sugars also play a primarily regulatory role in root architecture, interacting with auxin, ABA, ethylene and brassinolide. In addition, although glucose has been illustrated to antagonistically interact with ethylene during root growth, it has been determined to improve ethylene-induced root hair initiation and elongation [34]. In this study, WGCNA showed that two hormone–sucrose–root hair modules were linked by *IAA17*, and *SUS* was positioned in the center of the regulation network, co-expressed with *ARR4*, *TIFY10B*, *EBF1*, and *BSK3* in the hormone signal transduction pathway, *BGLH12* and *CMINV* in the sucrose synthesis pathway, and *IAA17*, a key gene related to root hair development (Figure 7 and Figure 8). The interwoven network between sugar and hormone signaling cascades plays a pivotal role in root hair architecture, which provides a new insight into the protection and breeding of endangered plants.

## 4. Conclusions

In summary, we firstly assembled the comparative transcriptomes for radicles with root hair (RH1) and radicles without root hair (RH0) of *A. beshanzuensis* test-tube plantlets and demonstrated that interactions of hormone and sugar signaling were primarily involved in the root hair morphogenesis of *A. beshanzuensis*. We hypothesized that root hair may be an important survival strategy, based on our study. Our findings provide a new insight into the mechanisms of the root hair morphogenesis of *A. beshanzuensis* and other endangered plants.

## 5. Materials and Methods

### 5.1. Plant Materials

The immature seeds were taken from the adult tree of *Abies beshanzuensis* grown in the National Nature Reserve of Baishanzu in Mt. Qingyuan, Lishui, Zhejiang Province in China (27°42′ N latitude, 119°11′ E longitude, 1775 m above sea level).

### 5.2. Immature Embryo Culture

Immature seeds from *A. beshanzuensis* were washed under running tap water for 3 h and sterilized in 70% analytical ethanol for 1 min. The seeds were then soaked in 0.1 wt% HgCl_2_ for 8 min before washing with sterile water 3-5 times. Outer seed coats of immature seeds were dissected under a stereomicroscope, and the isolated endosperm was inoculated into the medium of DCR (Gupta and Durzan Medium), coupled with sucrose (20 g·L^−1^), hydrolyze casein (500 mg·L^−1^) and Agar (8 g·L^−1^), pH 5.8. After inoculation, the cultures were incubated at (20 ± 2) °C for 3 days in the dark, and then incubated at low light for 10 days. We found that root hair developed on the cocked radicles (RH1), while nearly no root hair on the radicles attached to the medium (RH0), and this trait was maintained in vitro in plantlets. Different developmental stages of root (2 days before the root hair emerged, RH1/RH0_B; the day that root hair emerged, RH1/RH0_0; and 2 days after root hair emerged, RH1/RH0_A) were collected from immature embryo cultures, and placed immediately in liquid nitrogen, then stored at −80 °C for further RNA extraction and quantitative real-time PCR (qRT-PCR) analysis. Three biological replicates were performed.

### 5.3. RNA Sequencing and Data Analysis

Total RNA was isolated from eighteen samples using an EASY38 spin Plus Plant RNA Kit (Aidlab, Beijing, China) following the manufacturer’s protocol, and the integrity and purity of RNA was assessed as described by Liu et al. [67]. The Isoform Sequencing (Iso-Seq) library was prepared according to the Iso-Seq protocol using the Clontech SMARTer PCR cDNA Synthesis Kit and the BluePippin Size Selection System protocol as described by Pacific Biosciences (PN 100-092-800-03) to obtain the third-generation sequencing for a full-length transcriptome without a reference (PRJNA894800). Sequence data were processed using SMRTlink 5.0 software. Additional nucleotide errors in consensus reads were corrected using the Illumina RNA-seq data with the software LoRDEC. Cuffdiff(v2.1.1) was used to calculate FPKMs of all transcripts in each sample. Cuffdiff provides statistical routines for determining differential expression in digital transcript or gene expression data using a model based on the negative binomial distribution. Transcripts with a P-adjust < 0.05 were designated as differentially expressed. Differential expression analysis of the two groups was performed using the DESeq R package (1.18.0). Genes with an adjusted *p*-value < 0.05 found by DESeq were designated as differentially expressed (fold change >2-fold). Raw sequence data were uploaded to the National Center for Biotechnology Information (NCBI) Short Read Archive with the accession number PRJNA894826. Assembled unigenes were aligned to the Nr protein database of NCBI (http://www.ncbi.nlm.nih.gov (accessed on 5 November 2021)), KEGG pathway database (http://www.genom e.jp/kegg (accessed on 27 October 2022)), Swiss-Prot protein database (http://www.expasy.ch/sprot(accessed on 1 July 2020)), and COG database (http://www.ncbi.nlm.nih.gov/COG (accessed on 15 March 2021)). Blast2GO was used to produce the gene ontology (GO) analyses (http://www.geneontology.org/ (accessed on 1 January 2019)) for assembled unigenes. Heat maps were conducted with the ComplexHeatmap R package.

### 5.4. Network Analysis of Key Genes Involved in Root Hair Grow and Development in A. beshanzuensis

To identify the key genes involved in plant hormone signal transudation and sugar metabolism, which is responsible for the regulation of root hair growth and development, a system biology approach was applied using an R package for weighted gene co-expression network analysis (WGCNA), converting co-expression measures into connection weights [68] with a weighted cut-off value > 0.50. The co-expressed gene network was performed with Cytoscape software [69].

In a previous study, some root hair development genes were identified in *Arabidopsis thaliana*, such as *IAA17*, *XIK*, *VLN4*, *BHLH32*, *LRX3*, and *OXI1* [20,38]. These gene sequences were subjected to BLASTN searches against the transcriptome sequences to identify homologous sequences in *A. beshanzuensis* with E value < 10-10. The best blast homologous genes were used for WGCNA (Table S2).

### 5.5. Selection of Candidate Reference Genes

Seven candidate reference genes from *A. beshanzuensis* with a stable expression in the different root development stages were selected based on the transcriptome result (PRJNA894826) (Table S1). These genes are commonly regarded as housekeeping genes in some model plants. Their CDS sequences were blasted from the third-generation sequencing for a full-length transcriptome without a reference of *A. beshanzuensis* (PRJNA894800).

### 5.6. Quantitative Real-Time PCR (qRT-PCR) Validation

Total RNA was isolated, processed in accordance with the manufacturer’s protocol, and reverse-transcribed by a Prime Script RT reagent Kit (Takara, Dalian, China). Gene-specific primers (Table S1) were designed for the real-time quantitative PCR (qRT-PCR) determination of target genes by Primer Express3.0.1 (Applied Bio- systems). qRT-PCR was conducted with an ABI PRISM 7300 Real-Time PCR System (ABI, Foster City, CA, USA) using the SYBR Premix Ex Taq (Takara) coupled with an amplification procedure: 95 °C for 30 s, 40 cycles of 95 °C for 5 s and 60 °C for 34 s. Three biological and technical replicates were employed. The data were calculated using the 2^−ΔΔCT^ method [67,70,71].

## Data Availability

All data generated and analyzed in the study are included in this article and its Supplementary Materials. Additionally, the raw data were uploaded to the NCBI under accession numbers PRJNA894800 and PRJNA894826 (https://www.ncbi.nlm.nih.gov/bioproject (accessed on 27 October 2022)).

## Supplementary Materials

The following supporting information can be downloaded at: https://www.mdpi.com/article/10.3390/plants12020276/s1, Table S1: Primers used in the paper; Table S2: Homologous root hair development genes of *A. beshanzuensis* with the best alignment rate in *Arabidopsis thaliana;* Table S3: Modified concordant transcripts; Table S4: Length distribution statistics of transcripts after redundancy removal; Figure S1: GO assignments for functional classification of the assembled unigenes.
