# Supplementary material for: Comparative Transcriptome Analysis Reveals the Interaction of Sugar and Hormone Metabolism Involved in the Root Hair Morphogenesis of the Endangered Fir Abies beshanzuensis
Source: Plants (Basel). 2023 Jan 6;12(2):276. doi: 10.3390/plants12020276 (PMC9862426; doi:10.3390/plants12020276)
Supplement: Supplementary file 1 [file plants-12-00276-s001.zip › plants-2078789-supplementary.pdf]

Table S1. Primers used in the paper.

Primers name	Primers sequences (5'-3')
SUS1-f	GCCTGGTGTGTGGGATTATG
SUS1-r	GGAAAGGACGCATTGAAGGG
SUS2-f	GGGAAGAAGATTTTGCAGCCT
SUS2-r	TGGTGGAAGAATGATGGCCT
EBF1-1-f	GAGCCAGAGTCAAGGGAGTT
EBF1-1-r	TCCTCGACCACCCATACCTA
EBF1-2-f	ATGCCAACTCTTCTCAAGCG
EBF1-2-r	
ARR4-f	
ARR4-r	
BSK3-f	TCCCCATACAAGATGCAGCA GTGGCAGTAGTTCAAGCGAG AG-
BSK3-r	CAGCCTCTCGATGACTTT GCAGTTTGTGGAAGAAGCCT
SRK2E-f	GCCCATTCCATTGTCTGCTT GCACGTTTCTTCTTCCAGCA
SRK2E-r	ACAGATCTTGAGGCGAGGAG
CMINV1-f	GAGTTATTCGGTGCTGTGCC CAAGGGGCTGGATTTGGAAG CCCAAA-
CMINV1-r	GCATCACAGGCAAT GGCCCTCCATTCTTTGTAGC GATCGGTTTCTCTGG-
SPS2-2-f	CAAGC
SPS2-2-r	CGAAGATGGGAAGGGGCTAT GAAGAACACTTTGGCTGCA
TPS6-f	CGAGTGCAGAAGAGAGGTCA
TPS6-r	AAGGAAAGTTGCAGCACCA CCTTCACCATTGTCACCTGC
IAA17-f	CTGTCCAGAAAGCAGGGTCT
IAA17-r	GCATGGGCCGAAAATTCTCA
VLN4-1-f	TGGAGACGGACAAGATTGCT
VLN4-1-r	CAAGACAGTTTCAGCCGCAT
OXI1-f	ATTGTGTGAAAAGGCCAAGG
OXI1-r	TCAACATAGTCGCCCATGAA
XLK-f	AGCTGCAGAATGTGCCCTAT
XLK-r	CGCCTCATCGAAAACAAAAT
PP2A-f	AGGAACACCAGTTCGACCAC
PP2A-r	GTCGAGACTTCGACCACCTT
EIF2-f	TCTCGAACTTCCACAAAGCA
EIF2-r	GCGTGTGATTGAACGTTTTG
EIF3-f	GCAGCTCGAGAAGGTGATTC
EIF3-r	AATTCAGGGCTTTTGCATTG
EF1-f	ATCTTTGCTGGCAAGCAGTT
EF1-r	GCCAGTGAGGGTTTTAACGA
EIF-f	CTGGAATGGTTAAGGCTGGA
EIF-r	GGTCGATCGGGTACTTGAGA
UBQ-f	
UBQ-r	
act1-f	
act1-r	

Table S2. Homologous root hair development genes of *A. beshanzuensis* with the best alignment rate in *Arabidopsis thaliana.*

Gene annotation in Arabidopsis thaliana	*Arabidopsis thaliana* gene ID	Gene function in root hair development	Homologous genes in *Abies beshanzuensis*
BASIC HELIX-LOOP-HELIX 32 (BHLH32), TARGET OF MONOPTE-ROS 5 (TMO5)	AT3G25710	Changes in number; Changes in position	transcript_HQ_SEEDLING_transcript62398/f2p0/1885
ECTOPIC ROOT HAIR 1 (ERH1), ARABIDOPSIS INOSITOL PHOS-PHORYLCERAMIDE SYNTHASE 2 (ATIPCS2)	AT2G37940	Changes in position; Changes in number	transcript_HQ_SEEDLING_transcript70399/f3p0/1601
ECTOPIC ROOT HAIR 2 (ERH2), CHITINASE-LIKE PROTEIN 1 (CTL1), ECTOPIC DEPOSITION OF LIGNIN IN PITH 1	AT1G05850	Changes in number; Changes in position; Changes in length	transcript_HQ_SEEDLING_transcript72927/f9p0/1506
ECTOPIC ROOT HAIR 3 (ERH3), BOTERO 1 (BOT1), ECTOPIC ROOT HAIR 3 (ERH3), KATANIN 1 (KTN1), FURCA2 (FRC2), FAT ROOT (FTR)	AT1G80350	Changes in position	transcript_HQ_SEEDLING_transcript45116/f10p0/2301
ENHANCER OF GLABRA3 (EGL3), ATMYC-2	AT1G63650	Changes in number; Changes in position	transcript_HQ_SEEDLING_transcript31032/f2p0/2878
GLABRA3 (GL3), MYC6.2	AT5G41315	Changes in number; Changes in position	transcript_HQ_SEEDLING_transcript17960/f2p0/3461
SCRAMBLED (SCM), STRUBBELIG (SUB), STRUBBELIG-RECEPTOR FAMILY 9 (SRF9)	AT1G11130	Changes in position	transcript_HQ_SEEDLING_transcript27891/f5p0/2929
TRANSPARENT TESTA GLABRA1 (TTG1), UNARMED 23 (URM23)	AT5G24520	Changes in number; Changes in position	transcript_HQ_SEEDLING_transcript59148/f2p0/1979
AUXIN RESISTANT 3 (AXR3), IN-DOLE-3-ACETIC ACID INDUCIBLE 17 (IAA17)	AT1G04250	Root hairless	transcript_HQ_SEEDLING_transcript66121/f19p0/1751
TIP GROWTH DEFECTIVE1 (TIP1)	AT5G20350	Changes in length; Changes in shape; Branched root hairs	transcript_HQ_SEEDLING_transcript26029/f2p0/3037
1-PHOSPHATIDYLINOSITOL-4-PHOSPHATE 5-KINASE 3 (PIP5K3)	AT2G26420	Changes in length	transcript_HQ_SEEDLING_transcript7330/f2p0/4270
ACTIN INTERACTING PROTEIN 1 (AIP1-1)	AT2G01330	Changes in number; Changes in length	transcript_HQ_SEEDLING_transcript34450/f2p0/2731
ACTIN INTERACTING PROTEIN 1-2 (AIP1-2)	AT3G18060	Changes in number; Changes in length	transcript_HQ_SEEDLING_transcript30378/f26p0/2801
AGC2-1, OXIDATIVE SIGNAL-IN-DUCIBLE1 (OXI1)	AT3G25250	Changes in length	transcript_HQ_SEEDLING_transcript65552/f3p0/1792
ARABIDOPSIS K TRANSPORTER 1 (AKT1)	AT2G26650	Changes in length	transcript_HQ_SEEDLING_transcript19655/f3p0/3369
ARF-GAP DOMAIN 1 (AGD1)	AT5G61980	Changes in shape; changes in length	transcript_HQ_SEEDLING_transcript10719/f4p0/3914
ARP3, DISORTED1 (DIS1)	AT1G13180	Changes in shape	transcript_HQ_SEEDLING_transcript61648/f2p0/1899
AUXIN RESISTANT 1 (AXR1)	AT1G05180	Changes in length; Changes in number	transcript_HQ_SEEDLING_transcript52029/f2p0/2152
AUXIN TRANSPORTER PROTEIN 1 (AUX1), MODIFIER OF ARF7/NPH4 PHENOTYPES 1 (MAP1), WAVY ROOTS 5 (WAV5)	AT2G38120	Changes in length	transcript_HQ_SEEDLING_transcript45470/f2p0/2349
CAN OF WORMS1 (COW1), SHORT ROOT HAIR 1 (SRH1), ATSFH1	AT4G34580	Changes in length; Branched root hairs	transcript_HQ_SEEDLING_transcript42149/f2p0/2462
CELLULOSE SYNTHASE 6 (CESA6), ISOXABEN RESISTANT 2 (IXR2), PROCUSTE 1 (PRC1)	AT5G64740	Changes in length; changes in position	transcript_HQ_SEEDLING_transcript33428/f3p0/2758
COBRA-LIKE 9 (COBL9), SHAVEN 2 (SHV2), DEFORMED ROOT HAIRS 9 (DER9), MUTANT ROOT HAIR 4 (MRH4)	AT5G49270	Changes in length; Changes in shape	transcript_HQ_SEEDLING_transcript5521/f8p0/4476

ECTOPICALLY PARTING CELLS 1 (EPC1)	AT3G55830	Changes in length	transcript_HQ_SEEDLING_transcript76880/f2p0/1395
ETHYLENE OVERPRODUCER 1 (ETO1)	AT3G51770	Changes in length	transcript_HQ_SEEDLING_transcript10916/f11p0/3855
EXOCYST SUBUNIT EXO70 FAMILY PROTEIN A1 (EXO70A1)	AT5G03540	Changes in length	transcript_HQ_SEEDLING_transcript42527/f2p0/2452
EXPANSIN A7 (EXPA7)	AT1G12560	Changes in length	transcript_HQ_SEEDLING_transcript75333/f33p0/1368
HEXOKINASE-LIKE 1 (HKL1)	AT1G50460	Changes in shape; Changes in length	transcript_HQ_SEEDLING_transcript42923/f3p0/2456
INOSITOL POLYPHOSPHATE KINASE 2 ALPHA (IPK2A)	AT5G07370	Changes in length	transcript_HQ_SEEDLING_transcript2369/f3p0/5271
KEULE (KEU)	AT1G12360	Changes in shape; Changes in number	transcript_HQ_SEEDLING_transcript23380/f2p0/3169
KOJAK (KJK), CELLULOSE SYNTHASE LIKE D3 (CSLD3), ROOT HAIR DEFECTIVE 7 (RHD7)	AT3G03050	Changes in length; Changes in shape	transcript_HQ_SEEDLING_transcript3877/f2p0/4814
LEUCINE-RICH REPEAT/EXTENSIN 1 (LRX1)	AT1G12040	Changes in length; Changes in shape	transcript_HQ_SEEDLING_transcript35498/f2p0/2684
LJRHL1-LIKE 1 (LRL1), bHLH66	AT2G24260	Branched root hairs	transcript_HQ_SEEDLING_transcript30248/f2p0/2875
MAP KINASE 4 (MPK4)	AT4G01370	Changes in shape; Changes in position; Branched root hairs	transcript_HQ_SEEDLING_transcript64131/f5p0/1822
MYOSIN 2 (MYA2), ARABIDOPSIS MYOSIN 2 (ATMYA2), MYOSIN X1 2 (XI-2), MYOSIN XI-6 (XI-6)	AT5G43900	Changes in length	transcript_HQ_SEEDLING_transcript727/f5p0/6327
MYOSIN XI K (XIK)	AT5G20490	Changes in length	transcript_HQ_SEEDLING_transcript883/f16p0/6099
PHOSPATIDYLINOSITOL 3-KINASE (PI3K), VACUOLAR PROTEIN SORTING 34 (VPS34)	AT1G60490	Changes in length; Changes in number	transcript_HQ_SEEDLING_transcript19768/f2p0/3359
PROFILIN 1, PFN1, PRF1	AT2G19760	Changes in length	transcript_HQ_SEEDLING_transcript81488/f10p0/986
PROLINE-RICH EXTENSIN-LIKE RECEPTOR KINASE 13 (PERK13), ROOT HAIR SPECIFIC 10 (RHS10)	AT1G70460	Changes in length	transcript_HQ_SEEDLING_transcript13228/f2p0/3745
PROLYL 4-HYDROXYLASE 2 (P4H2)	AT3G06300	Changes in length; Changes in number	transcript_HQ_SEEDLING_transcript70223/f2p0/1644
PROLYL 4-HYDROXYLASE 5 (P4H5)	AT2G17720	Changes in length	transcript_HQ_SEEDLING_transcript60028/f3p0/1932
REDUCED RESIDUAL ARABINOSE 1 (RRA1)	AT1G75120	Changes in length	transcript_HQ_SEEDLING_transcript57329/f2p0/2004
RHO GUANYL-NUCLEOTIDE EXCHANGE FACTOR 4 (ROPGEF4), ROOT HAIR SPECIFIC 11 (RHS11)	AT2G45890	Changes in length	transcript_HQ_SEEDLING_transcript37808/f2p0/2606
RHO-RELATED PROTEIN FROM PLANTS 4 (ROP4), RAC-LIKE GTP BINDING PROTEIN 5 (RAC5), ARABIDOPSIS THALIANA GERANYLGERANYLATED PROTEIN 3 (ATGP3)	AT1G75840	Changes in shape	transcript_HQ_SEEDLING_transcript80501/f5p0/1094
ROOT HAIR DEFECTIVE 1 (RHD1), ROOT EPIDERMAL BULGER (REB1), UDP-GLUCOSE 4-EPIMERASE (UGE4)	AT1G64440	Changes in length; Changes in shape	transcript_HQ_SEEDLING_transcript70343/f4p0/1646
ROOT HAIR DEFECTIVE 2 (RHD2), RESPIRATORY BURST OXIDASE HOMOLOG C (RBOHC)	AT5G51060	Changes in length	transcript_HQ_SEEDLING_transcript21176/f5p0/3277
ROOT HAIR DEFECTIVE 3 (RHD3), GOLGI MUTANT 8 (GOM8)	AT3G13870	Changes in length; Changes in shape ; Branched root hairs	transcript_HQ_SEEDLING_transcript15244/f2p0/3633
ROOT HAIR DEFECTIVE 4 (RHD4), ATSAC7	AT3G51460	Changes in length; Changes in shape	transcript_HQ_SEEDLING_transcript28888/f2p0/2943
STOMATAL CYTOKINESIS-DEFECTIVE 1 (SCD1)	AT1G49040	Changes in length	transcript_HQ_SEEDLING_transcript6096/f3p0/4416
SUPPRESSOR OF AUXIN RESISTANCE1 (SAR1), ARABIDOPSIS NUCLEOPORIN 160 (ATNUP160), NUCLEOPORIN 160 (NUP160)	AT1G33410	Changes in shape; Changes in number	transcript_HQ_SEEDLING_transcript2075/f6p0/5339

VILLIN 4 (VLN4)	AT4G30160	Changes in length	transcript_HQ_SEEDLING_transcript25203/f19p0/3003
XYLOGLUCAN XYLOSYLTRANS-FERASE 5 (XXT5)	AT1G74380	Changes in shape	transcript_HQ_SEEDLING_transcript13703/f4p0/3689

Table S3. Modified concordant transcripts.

Sample	Consensus_number	Min_length	Max_length	Mean_length	N50
SEEDLING	83384	79	12353	2660	2939

Table S4. Length distribution statistics of transcripts after redundancy removal.

Sample	Total_nucleotides	Total_number	Mean_length	Min_length	Max_length	N50	N90
SEEDLING	75768368	28923	2620	226	12339	2918	1672

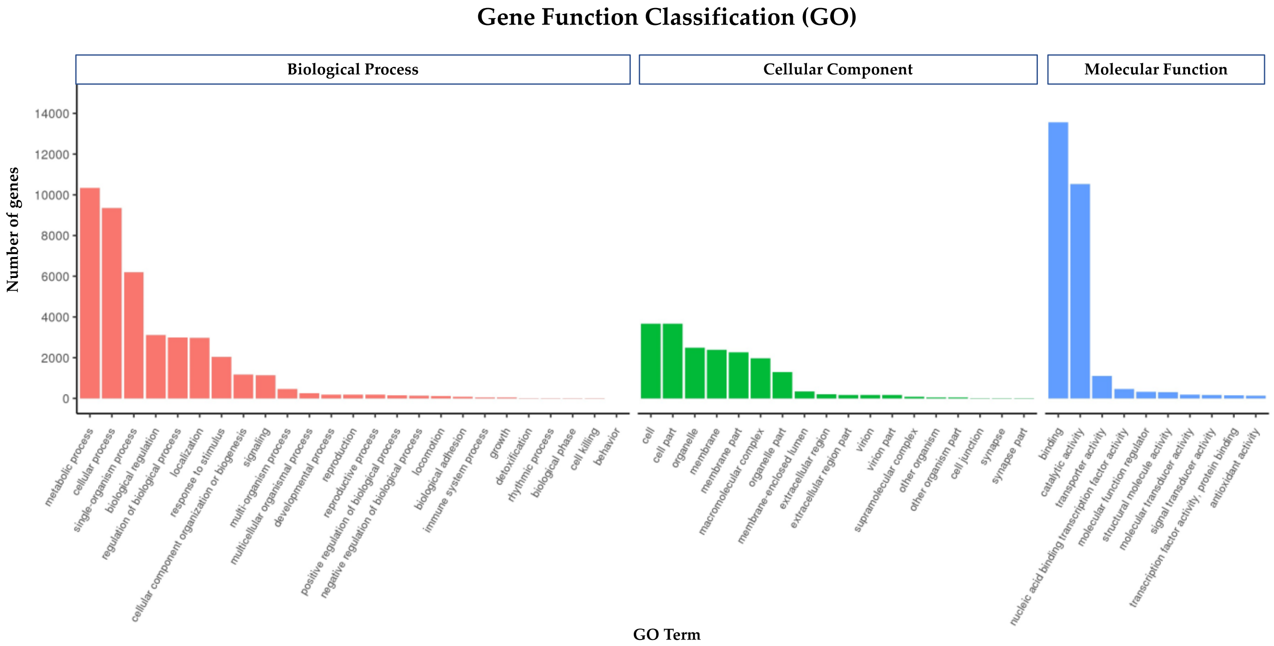

Figure S1. GO assignments for functional classification of the assembled unigenes.
